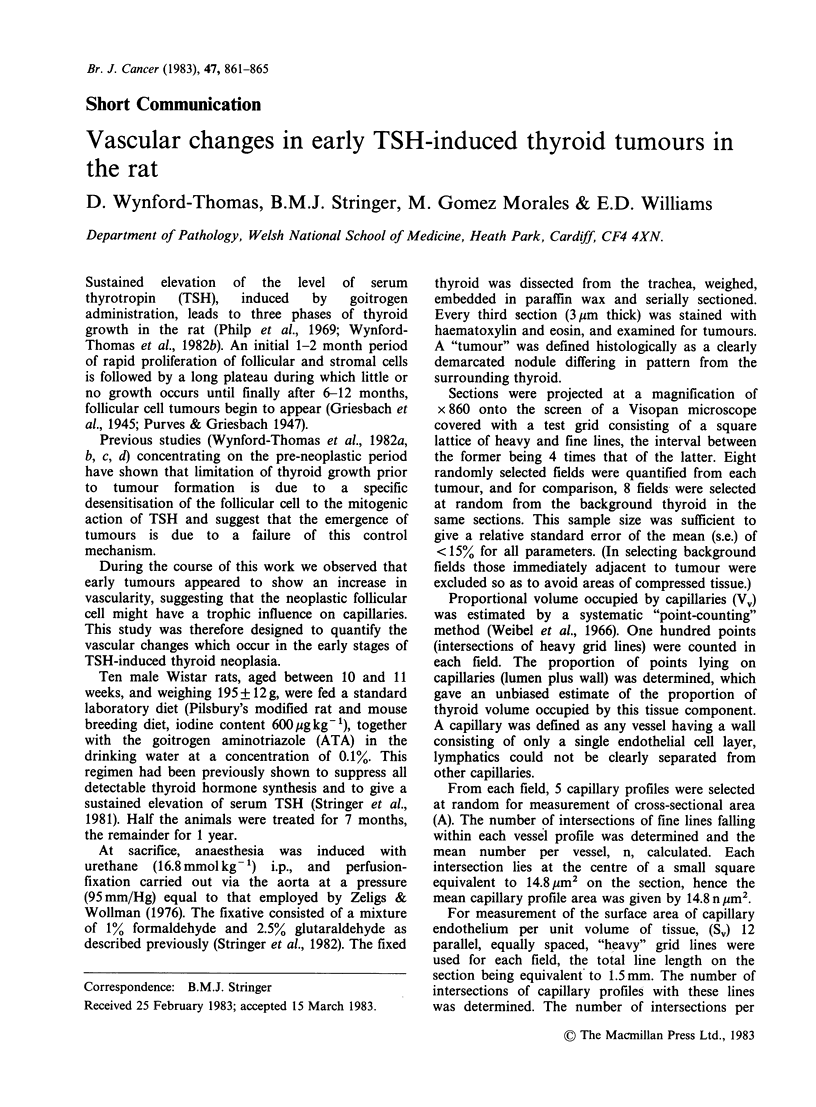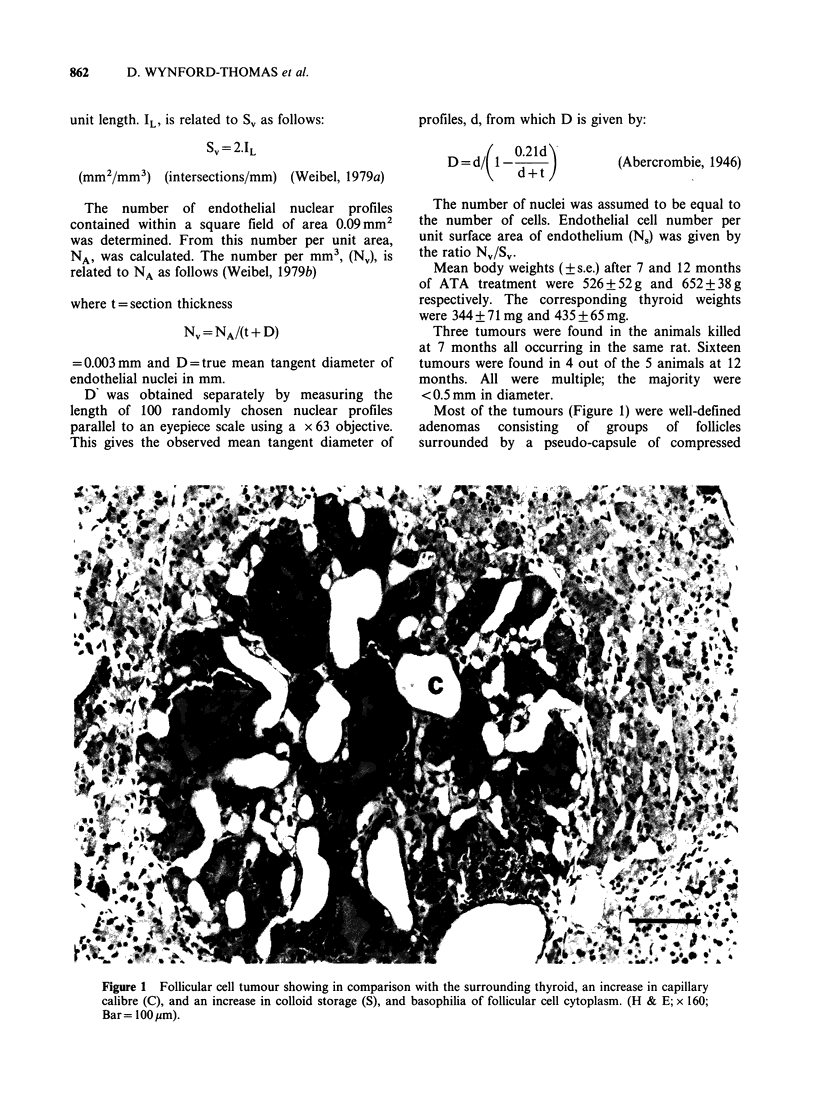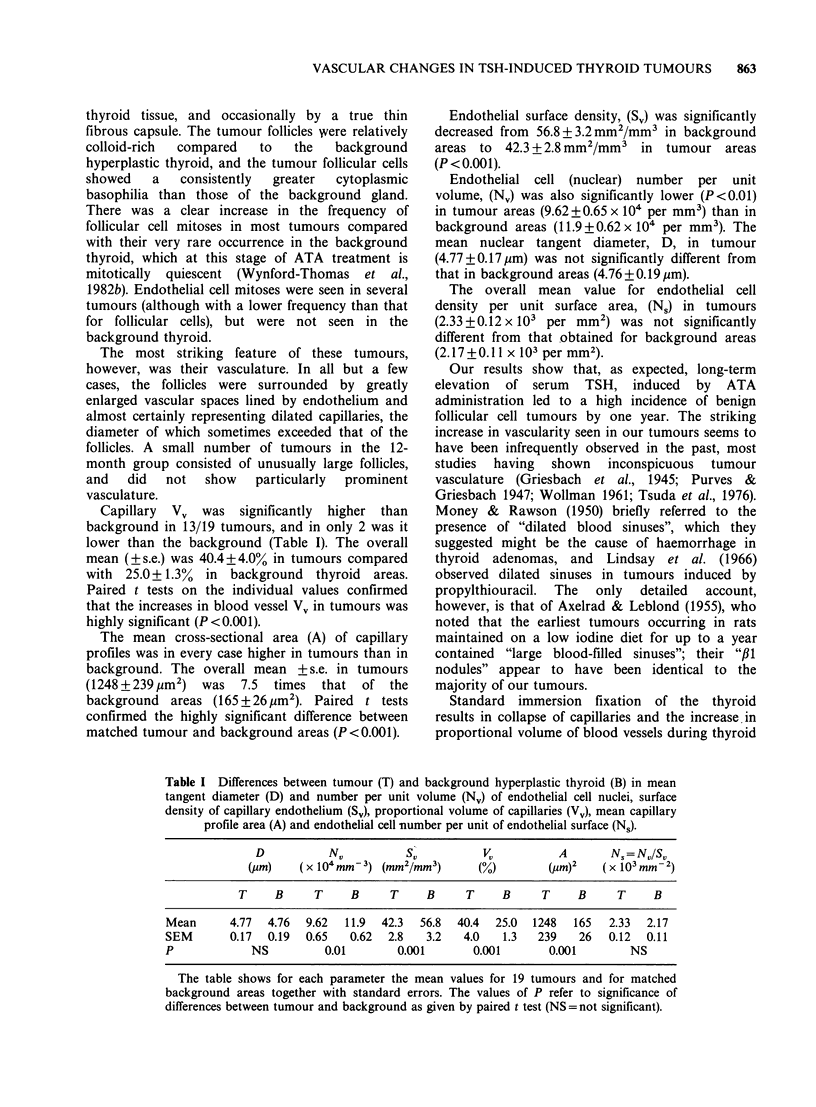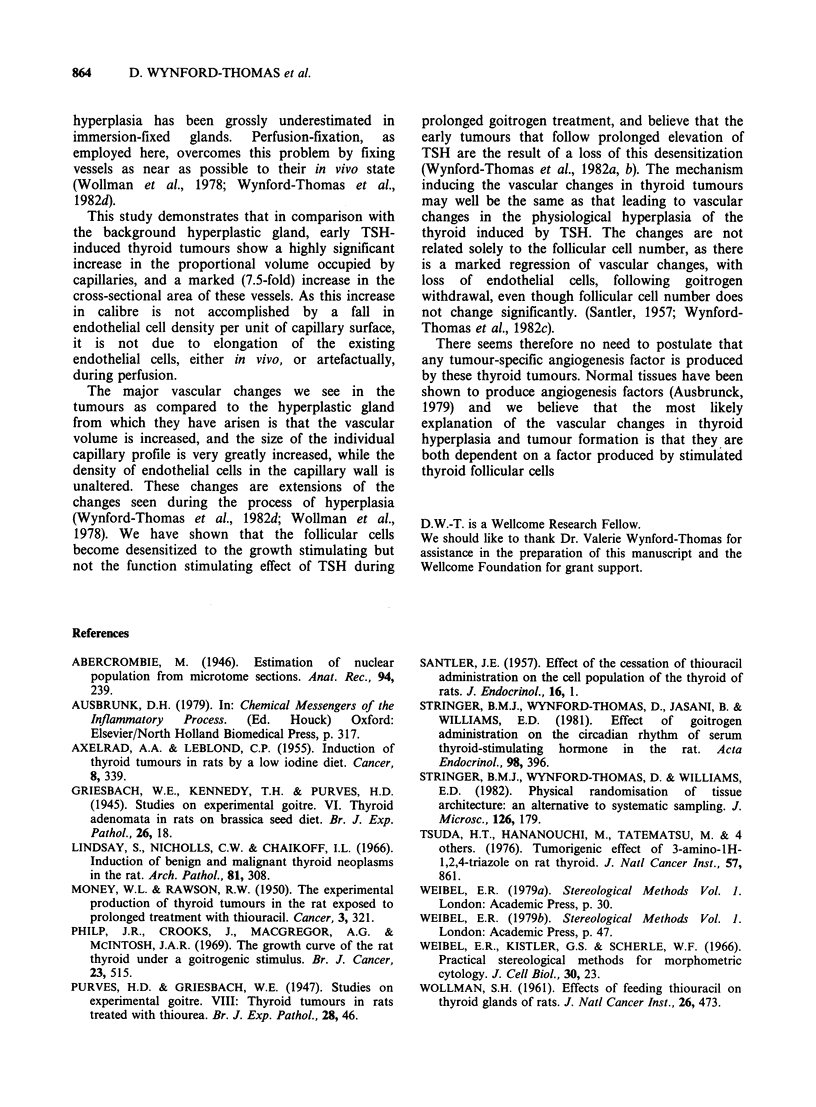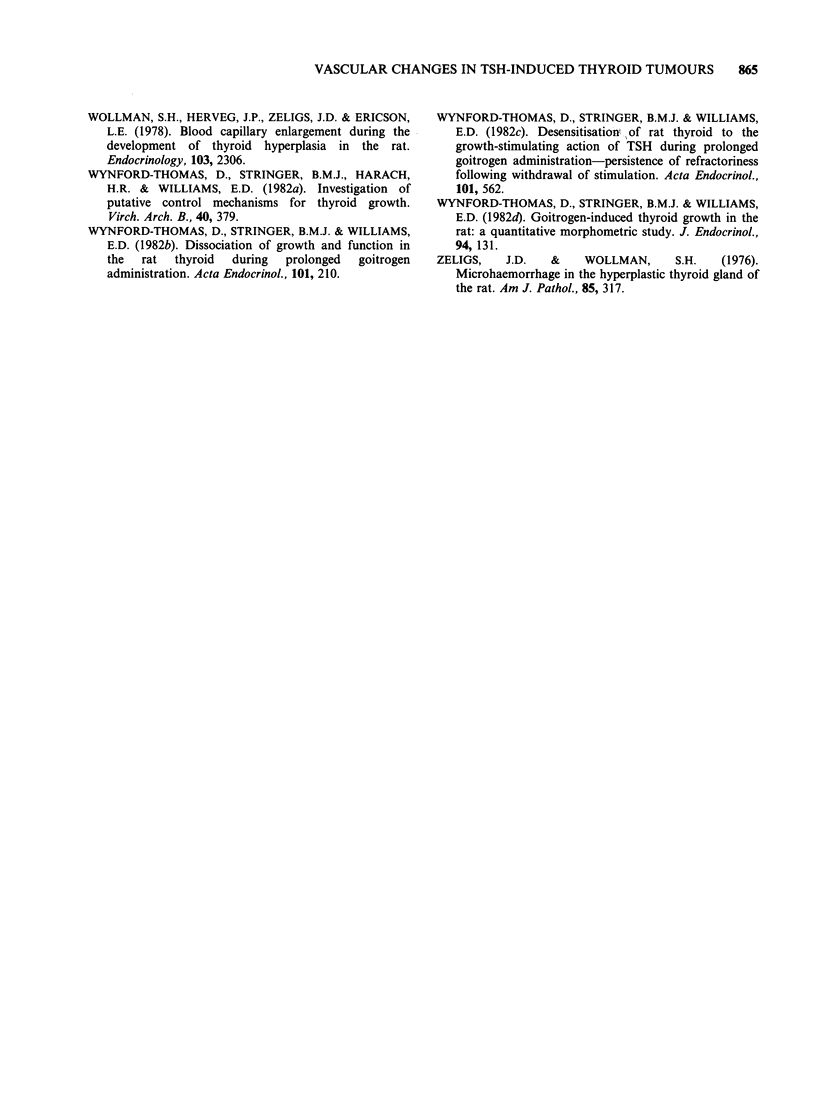# Vascular changes in early TSH-induced thyroid tumours in the rat.

**DOI:** 10.1038/bjc.1983.143

**Published:** 1983-06

**Authors:** D. Wynford-Thomas, B. M. Stringer, M. Gomez Morales, E. D. Williams

## Abstract

**Images:**


*Br. J. Cancer* (1983), **47**, 861–865

### Short Communication

### Vascular changes in early TSH-induced thyroid tumours in the rat

D. Wynford-Thomas, B.M.J. Stringer, M. Gomez Morales & E.D. Williams

*Department of Pathology, Welsh National School of Medicine, Heath Park, Cardiff, CF4 4XN.*

Sustained elevation of the level of serum thyrotropin (TSH), induced by goitrogen administration, leads to three phases of thyroid growth in the rat (Philp *et al.*, 1969; Wynford-Thomas *et al.*, 1982*b*). An initial 1–2 month period of rapid proliferation of follicular and stromal cells is followed by a long plateau during which little or no growth occurs until finally after 6–12 months, follicular cell tumours begin to appear (Griesbach *et al.*, 1945; Purves & Griesbach 1947).

Previous studies (Wynford-Thomas *et al.*, 1982*a*, *b*, *c*, *d*) concentrating on the pre-neoplastic period have shown that limitation of thyroid growth prior to tumour formation is due to a specific desensitisation of the follicular cell to the mitogenic action of TSH and suggest that the emergence of tumours is due to a failure of this control mechanism.

During the course of this work we observed that early tumours appeared to show an increase in vascularity, suggesting that the neoplastic follicular cell might have a trophic influence on capillaries. This study was therefore designed to quantify the vascular changes which occur in the early stages of TSH-induced thyroid neoplasia.

Ten male Wistar rats, aged between 10 and 11 weeks, and weighing $195 \pm 12$ g, were fed a standard laboratory diet (Pilsbury's modified rat and mouse breeding diet, iodine content $600\,\mu g\,kg^{-1}$), together with the goitrogen aminotriazole (ATA) in the drinking water at a concentration of 0.1%. This regimen had been previously shown to suppress all detectable thyroid hormone synthesis and to give a sustained elevation of serum TSH (Stringer *et al.*, 1981). Half the animals were treated for 7 months, the remainder for 1 year.

At sacrifice, anaesthesia was induced with urethane ($16.8\,mmol\,kg^{-1}$) i.p., and perfusion-fixation carried out via the aorta at a pressure (95 mm/Hg) equal to that employed by Zeligs & Wollman (1976). The fixative consisted of a mixture of 1% formaldehyde and 2.5% glutaraldehyde as described previously (Stringer *et al.*, 1982). The fixed thyroid was dissected from the trachea, weighed, embedded in paraffin wax and serially sectioned. Every third section ($3\,\mu m$ thick) was stained with haematoxylin and eosin, and examined for tumours. A "tumour" was defined histologically as a clearly demarcated nodule differing in pattern from the surrounding thyroid.

Sections were projected at a magnification of ×860 onto the screen of a Visopan microscope covered with a test grid consisting of a square lattice of heavy and fine lines, the interval between the former being 4 times that of the latter. Eight randomly selected fields were quantified from each tumour, and for comparison, 8 fields were selected at random from the background thyroid in the same sections. This sample size was sufficient to give a relative standard error of the mean (s.e.) of <15% for all parameters. (In selecting background fields those immediately adjacent to tumour were excluded so as to avoid areas of compressed tissue.)

Proportional volume occupied by capillaries ($V_v$) was estimated by a systematic "point-counting" method (Weibel *et al.*, 1966). One hundred points (intersections of heavy grid lines) were counted in each field. The proportion of points lying on capillaries (lumen plus wall) was determined, which gave an unbiased estimate of the proportion of thyroid volume occupied by this tissue component. A capillary was defined as any vessel having a wall consisting of only a single endothelial cell layer, lymphatics could not be clearly separated from other capillaries.

From each field, 5 capillary profiles were selected at random for measurement of cross-sectional area (A). The number of intersections of fine lines falling within each vessel profile was determined and the mean number per vessel, n, calculated. Each intersection lies at the centre of a small square equivalent to $14.8\,\mu m^2$ on the section, hence the mean capillary profile area was given by $14.8\,n\,\mu m^2$.

For measurement of the surface area of capillary endothelium per unit volume of tissue, ($S_v$) 12 parallel, equally spaced, "heavy" grid lines were used for each field, the total line length on the section being equivalent to 1.5 mm. The number of intersections of capillary profiles with these lines was determined. The number of intersections per

Correspondence: B.M.J. Stringer

Received 25 February 1983; accepted 15 March 1983.

© The Macmillan Press Ltd., 1983

unit length. $I_L$, is related to $S_v$ as follows:

$$S_v = 2.I_L$$

($mm^2/mm^3$) (intersections/mm) (Weibel, 1979*a*)

The number of endothelial nuclear profiles contained within a square field of area $0.09\,mm^2$ was determined. From this number per unit area, $N_A$, was calculated. The number per $mm^3$, ($N_v$), is related to $N_A$ as follows (Weibel, 1979*b*)

where t = section thickness

$$N_v = N_A/(t + D)$$

= 0.003 mm and D = true mean tangent diameter of endothelial nuclei in mm.

D' was obtained separately by measuring the length of 100 randomly chosen nuclear profiles parallel to an eyepiece scale using a × 63 objective. This gives the observed mean tangent diameter of profiles, d, from which D is given by:

$$D = d/\left(1 - \frac{0.21d'}{d + t}\right) \quad \text{(Abercrombie, 1946)}$$

The number of nuclei was assumed to be equal to the number of cells. Endothelial cell number per unit surface area of endothelium ($N_s$) was given by the ratio $N_v/S_v$.

Mean body weights (±s.e.) after 7 and 12 months of ATA treatment were 526±52 g and 652±38 g respectively. The corresponding thyroid weights were 344±71 mg and 435±65 mg.

Three tumours were found in the animals killed at 7 months all occurring in the same rat. Sixteen tumours were found in 4 out of the 5 animals at 12 months. All were multiple; the majority were <0.5 mm in diameter.

Most of the tumours (Figure 1) were well-defined adenomas consisting of groups of follicles surrounded by a pseudo-capsule of compressed

**Figure 1** Follicular cell tumour showing in comparison with the surrounding thyroid, an increase in capillary calibre (C), and an increase in colloid storage (S), and basophilia of follicular cell cytoplasm. (H & E; × 160; Bar = 100 μm).

thyroid tissue, and occasionally by a true thin fibrous capsule. The tumour follicles were relatively colloid-rich compared to the background hyperplastic thyroid, and the tumour follicular cells showed a consistently greater cytoplasmic basophilia than those of the background gland. There was a clear increase in the frequency of follicular cell mitoses in most tumours compared with their very rare occurrence in the background thyroid, which at this stage of ATA treatment is mitotically quiescent (Wynford-Thomas *et al.*, 1982*b*). Endothelial cell mitoses were seen in several tumours (although with a lower frequency than that for follicular cells), but were not seen in the background thyroid.

The most striking feature of these tumours, however, was their vasculature. In all but a few cases, the follicles were surrounded by greatly enlarged vascular spaces lined by endothelium and almost certainly representing dilated capillaries, the diameter of which sometimes exceeded that of the follicles. A small number of tumours in the 12-month group consisted of unusually large follicles, and did not show particularly prominent vasculature.

Capillary $V_v$ was significantly higher than background in 13/19 tumours, and in only 2 was it lower than the background (Table I). The overall mean ($\pm$s.e.) was $40.4 \pm 4.0\%$ in tumours compared with $25.0 \pm 1.3\%$ in background thyroid areas. Paired $t$ tests on the individual values confirmed that the increases in blood vessel $V_v$ in tumours was highly significant ($P < 0.001$).

The mean cross-sectional area (A) of capillary profiles was in every case higher in tumours than in background. The overall mean $\pm$s.e. in tumours ($1248 \pm 239\,\mu m^2$) was 7.5 times that of the background areas ($165 \pm 26\,\mu m^2$). Paired $t$ tests confirmed the highly significant difference between matched tumour and background areas ($P < 0.001$).

Endothelial surface density, ($S_v$) was significantly decreased from $56.8 \pm 3.2\,mm^2/mm^3$ in background areas to $42.3 \pm 2.8\,mm^2/mm^3$ in tumour areas ($P < 0.001$).

Endothelial cell (nuclear) number per unit volume, ($N_v$) was also significantly lower ($P < 0.01$) in tumour areas ($9.62 \pm 0.65 \times 10^4$ per $mm^3$) than in background areas ($11.9 \pm 0.62 \times 10^4$ per $mm^3$). The mean nuclear tangent diameter, D, in tumour ($4.77 \pm 0.17\,\mu m$) was not significantly different from that in background areas ($4.76 \pm 0.19\,\mu m$).

The overall mean value for endothelial cell density per unit surface area, ($N_s$) in tumours ($2.33 \pm 0.12 \times 10^3$ per $mm^2$) was not significantly different from that obtained for background areas ($2.17 \pm 0.11 \times 10^3$ per $mm^2$).

Our results show that, as expected, long-term elevation of serum TSH, induced by ATA administration led to a high incidence of benign follicular cell tumours by one year. The striking increase in vascularity seen in our tumours seems to have been infrequently observed in the past, most studies having shown inconspicuous tumour vasculature (Griesbach *et al.*, 1945; Purves & Griesbach 1947; Wollman 1961; Tsuda *et al.*, 1976). Money & Rawson (1950) briefly referred to the presence of "dilated blood sinuses", which they suggested might be the cause of haemorrhage in thyroid adenomas, and Lindsay *et al.* (1966) observed dilated sinuses in tumours induced by propylthiouracil. The only detailed account, however, is that of Axelrad & Leblond (1955), who noted that the earliest tumours occurring in rats maintained on a low iodine diet for up to a year contained "large blood-filled sinuses"; their "$\beta$1 nodules" appear to have been identical to the majority of our tumours.

Standard immersion fixation of the thyroid results in collapse of capillaries and the increase in proportional volume of blood vessels during thyroid

**Table I** Differences between tumour (T) and background hyperplastic thyroid (B) in mean tangent diameter (D) and number per unit volume ($N_V$) of endothelial cell nuclei, surface density of capillary endothelium ($S_V$), proportional volume of capillaries ($V_V$), mean capillary profile area (A) and endothelial cell number per unit of endothelial surface ($N_S$).

| | $D$ ($\mu m$) | | $N_v$ ($\times 10^4 mm^{-3}$) | | $S_v$ ($mm^2/mm^3$) | | $V_v$ (%) | | $A$ ($\mu m)^2$ | | $N_s = N_v/S_v$ ($\times 10^3 mm^{-2}$) | |
|---|---|---|---|---|---|---|---|---|---|---|---|---|
| | *T* | *B* | *T* | *B* | *T* | *B* | *T* | *B* | *T* | *B* | *T* | *B* |
| Mean | 4.77 | 4.76 | 9.62 | 11.9 | 42.3 | 56.8 | 40.4 | 25.0 | 1248 | 165 | 2.33 | 2.17 |
| SEM | 0.17 | 0.19 | 0.65 | 0.62 | 2.8 | 3.2 | 4.0 | 1.3 | 239 | 26 | 0.12 | 0.11 |
| *P* | NS | | 0.01 | | 0.001 | | 0.001 | | 0.001 | | NS | |

The table shows for each parameter the mean values for 19 tumours and for matched background areas together with standard errors. The values of $P$ refer to significance of differences between tumour and background as given by paired $t$ test (NS = not significant).

hyperplasia has been grossly underestimated in immersion-fixed glands. Perfusion-fixation, as employed here, overcomes this problem by fixing vessels as near as possible to their *in vivo* state (Wollman *et al.*, 1978; Wynford-Thomas *et al.*, 1982*d*).

This study demonstrates that in comparison with the background hyperplastic gland, early TSH-induced thyroid tumours show a highly significant increase in the proportional volume occupied by capillaries, and a marked (7.5-fold) increase in the cross-sectional area of these vessels. As this increase in calibre is not accomplished by a fall in endothelial cell density per unit of capillary surface, it is not due to elongation of the existing endothelial cells, either *in vivo*, or artefactually, during perfusion.

The major vascular changes we see in the tumours as compared to the hyperplastic gland from which they have arisen is that the vascular volume is increased, and the size of the individual capillary profile is very greatly increased, while the density of endothelial cells in the capillary wall is unaltered. These changes are extensions of the changes seen during the process of hyperplasia (Wynford-Thomas *et al.*, 1982*d*; Wollman *et al.*, 1978). We have shown that the follicular cells become desensitized to the growth stimulating but not the function stimulating effect of TSH during prolonged goitrogen treatment, and believe that the early tumours that follow prolonged elevation of TSH are the result of a loss of this desensitization (Wynford-Thomas *et al.*, 1982*a*, *b*). The mechanism inducing the vascular changes in thyroid tumours may well be the same as that leading to vascular changes in the physiological hyperplasia of the thyroid induced by TSH. The changes are not related solely to the follicular cell number, as there is a marked regression of vascular changes, with loss of endothelial cells, following goitrogen withdrawal, even though follicular cell number does not change significantly. (Santler, 1957; Wynford-Thomas *et al.*, 1982*c*).

There seems therefore no need to postulate that any tumour-specific angiogenesis factor is produced by these thyroid tumours. Normal tissues have been shown to produce angiogenesis factors (Ausbrunck, 1979) and we believe that the most likely explanation of the vascular changes in thyroid hyperplasia and tumour formation is that they are both dependent on a factor produced by stimulated thyroid follicular cells

D.W.-T. is a Wellcome Research Fellow.

We should like to thank Dr. Valerie Wynford-Thomas for assistance in the preparation of this manuscript and the Wellcome Foundation for grant support.

### References

ABERCROMBIE, M. (1946). Estimation of nuclear population from microtome sections. *Anat. Rec.*, **94**, 239.

AUSBRUNK, D.H. (1979). In: *Chemical Messengers of the Inflammatory Process*. (Ed. Houck) Oxford: Elsevier/North Holland Biomedical Press, p. 317.

AXELRAD, A.A. & LEBLOND, C.P. (1955). Induction of thyroid tumours in rats by a low iodine diet. *Cancer*, **8**, 339.

GRIESBACH, W.E., KENNEDY, T.H. & PURVES, H.D. (1945). Studies on experimental goitre. VI. Thyroid adenomata in rats on brassica seed diet. *Br. J. Exp. Pathol.*, **26**, 18.

LINDSAY, S., NICHOLLS, C.W. & CHAIKOFF, I.L. (1966). Induction of benign and malignant thyroid neoplasms in the rat. *Arch. Pathol.*, **81**, 308.

MONEY, W.L. & RAWSON, R.W. (1950). The experimental production of thyroid tumours in the rat exposed to prolonged treatment with thiouracil. *Cancer*, **3**, 321.

PHILP, J.R., CROOKS, J., MACGREGOR, A.G. & MCINTOSH, J.A.R. (1969). The growth curve of the rat thyroid under a goitrogenic stimulus. *Br. J. Cancer*, **23**, 515.

PURVES, H.D. & GRIESBACH, W.E. (1947). Studies on experimental goitre. VIII: Thyroid tumours in rats treated with thiourea. *Br. J. Exp. Pathol.*, **28**, 46.

SANTLER, J.E. (1957). Effect of the cessation of thiouracil administration on the cell population of the thyroid of rats. *J. Endocrinol.*, **16**, 1.

STRINGER, B.M.J., WYNFORD-THOMAS, D., JASANI, B. & WILLIAMS, E.D. (1981). Effect of goitrogen administration on the circadian rhythm of serum thyroid-stimulating hormone in the rat. *Acta Endocrinol.*, **98**, 396.

STRINGER, B.M.J., WYNFORD-THOMAS, D. & WILLIAMS, E.D. (1982). Physical randomisation of tissue architecture: an alternative to systematic sampling. *J. Microsc.*, **126**, 179.

TSUDA, H.T., HANANOUCHI, M., TATEMATSU, M. & 4 others. (1976). Tumorigenic effect of 3-amino-1H-1,2,4-triazole on rat thyroid. *J. Natl Cancer Inst.*, **57**, 861.

WEIBEL, E.R. (1979*a*). *Stereological Methods Vol. 1*. London: Academic Press, p. 30.

WEIBEL, E.R. (1979*b*). *Stereological Methods Vol. 1*. London: Academic Press, p. 47.

WEIBEL, E.R., KISTLER, G.S. & SCHERLE, W.F. (1966). Practical stereological methods for morphometric cytology. *J. Cell Biol.*, **30**, 23.

WOLLMAN, S.H. (1961). Effects of feeding thiouracil on thyroid glands of rats. *J. Natl Cancer Inst.*, **26**, 473.

WOLLMAN, S.H., HERVEG, J.P., ZELIGS, J.D. & ERICSON, L.E. (1978). Blood capillary enlargement during the development of thyroid hyperplasia in the rat. *Endocrinology*, **103**, 2306.

WYNFORD-THOMAS, D., STRINGER, B.M.J., HARACH, H.R. & WILLIAMS, E.D. (1982*a*). Investigation of putative control mechanisms for thyroid growth. *Virch. Arch. B.*, **40**, 379.

WYNFORD-THOMAS, D., STRINGER, B.M.J. & WILLIAMS, E.D. (1982*b*). Dissociation of growth and function in the rat thyroid during prolonged goitrogen administration. *Acta Endocrinol.*, **101**, 210.

WYNFORD-THOMAS, D., STRINGER, B.M.J. & WILLIAMS, E.D. (1982*c*). Desensitisation of rat thyroid to the growth-stimulating action of TSH during prolonged goitrogen administration—persistence of refractoriness following withdrawal of stimulation. *Acta Endocrinol.*, **101**, 562.

WYNFORD-THOMAS, D., STRINGER, B.M.J. & WILLIAMS, E.D. (1982*d*). Goitrogen-induced thyroid growth in the rat: a quantitative morphometric study. *J. Endocrinol.*, **94**, 131.

ZELIGS, J.D. & WOLLMAN, S.H. (1976). Microhaemorrhage in the hyperplastic thyroid gland of the rat. *Am J. Pathol.*, **85**, 317.